# Self-assembling ferritin-dendrimer nanoparticles for targeted delivery of nucleic acids to myeloid leukemia cells

**DOI:** 10.1186/s12951-021-00921-5

**Published:** 2021-06-09

**Authors:** Federica Palombarini, Silvia Masciarelli, Alessio Incocciati, Francesca Liccardo, Elisa Di Fabio, Antonia Iazzetti, Giancarlo Fabrizi, Francesco Fazi, Alberto Macone, Alessandra Bonamore, Alberto Boffi

**Affiliations:** 1 Department of Biochemical Sciences “Alessandro Rossi Fanelli”, Sapienza University of Rome, Piazzale Aldo Moro 5, 00185 Rome, Italy; 2 Department of Anatomical, Histological, Forensic & Orthopaedic Sciences, Section of Histology and Medical Embryology, Sapienza University of Rome, Laboratory Affiliated To Istituto Pasteur Italia-Fondazione Cenci Bolognetti, Via A. Scarpa, 14-16, 00161 Rome, Italy; 3 Department of Chemistry and Technology of Drugs, Sapienza University of Rome, Piazzale Aldo Moro 5, 00185 Rome, Italy; 4 Histology and Embryology Section, Department of Life Science and Public Health, Fondazione Policlinico Universitario A. Gemelli IRCCS, Largo Agostino Gemelli 8, 00168 Roma, Italy; 5 Center for Life Nano Science@Sapienza, Istituto Italiano Di Tecnologia, V.le Regina Elena 291, 00161 Rome, Italy

**Keywords:** Ferritin, Protein nanoparticles, Self-assembly, Targeted delivery, Dendrimers, PAMAM, miRNA

## Abstract

**Background:**

In recent years, the use of ferritins as nano-vehicles for drug delivery is taking center stage. Compared to other similar nanocarriers, *Archaeoglobus fulgidus* ferritin is particularly interesting due to its unique ability to assemble-disassemble under very mild conditions. Recently this ferritin was engineered to get a chimeric protein targeted to human CD71 receptor, typically overexpressed in cancer cells.

**Results:**

*Archaeoglobus fulgidus* chimeric ferritin was used to generate a self-assembling hybrid nanoparticle hosting an aminic dendrimer together with a small nucleic acid. The positively charged dendrimer can indeed establish electrostatic interactions with the chimeric ferritin internal surface, allowing the formation of a protein-dendrimer binary system. The 4 large triangular openings on the ferritin shell represent a gate for negatively charged small RNAs, which access the internal cavity attracted by the dense positive charge of the dendrimer. This ternary protein-dendrimer-RNA system is efficiently uptaken by acute myeloid leukemia cells, typically difficult to transfect. As a proof of concept, we used a microRNA whose cellular delivery and induced phenotypic effects can be easily detected. In this article we have demonstrated that this hybrid nanoparticle successfully delivers a pre-miRNA to leukemia cells. Once delivered, the nucleic acid is released into the cytosol and processed to mature miRNA, thus eliciting phenotypic effects and morphological changes similar to the initial stages of granulocyte differentiation.

**Conclusion:**

The results here presented pave the way for the design of a new family of protein-based transfecting agents that can specifically target a wide range of diseased cells.

**Graphic abstract:**

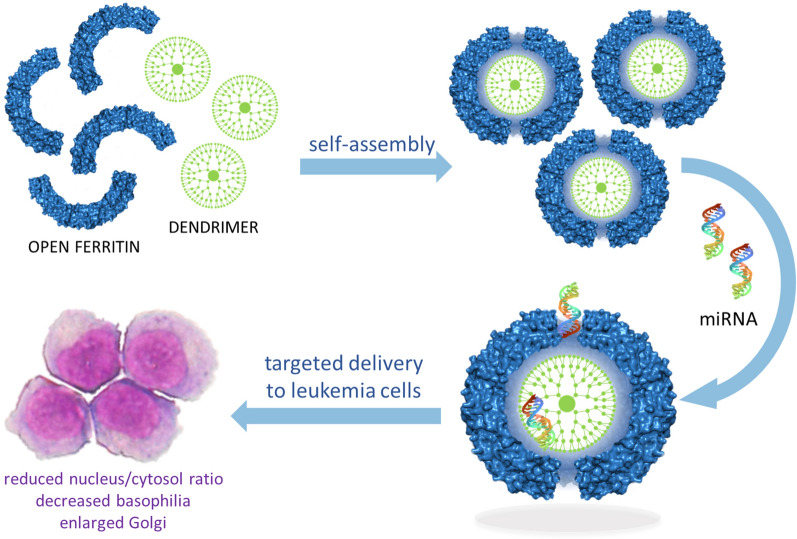

**Supplementary Information:**

The online version contains supplementary material available at 10.1186/s12951-021-00921-5.

## Introduction

The development of nanosystems for drug delivery is a fast-expanding area in scientific research, especially in the field of tumor treatment [1–8]. Among all nanosystems, protein nanoparticles occupy a privileged place due to their multiple properties, including biocompatibility, uniformity of size and shape, and the ease of production in the recombinant form [9–11]. Accordingly, ferritins are among the most studied protein nanosystems. They typically display a quaternary structure characterized by a hollow spherical cage with an internal cavity large enough to host different cargoes including drugs, imaging agents, small proteins, and nucleic acids [12–18]. Special attention is currently focused on human H ferritin for its ability to recognize the CD71 receptor, typically overexpressed in cancer cells [19]. In general, cargoes are incorporated into human ferritin by disassembling the protein at acidic or basic pH values and reassembling the 24-subunit oligomer (24-mer) under neutral conditions [20]. However, the pH jump method suffers from low yields of reconstituted protein as well as instability of many bioactive molecules at extreme pH values [21]. Recently, our research team developed a recombinant chimeric ferritin capable of disassembling/reassembling under very mild conditions, thus allowing safe and high yield incorporation of the selected cargo. This protein originates from *Archaeoglobus fulgidus* ferritin (AfFt) endowed with a unique cation-dependent polymerization equilibrium in which a dissociated dimeric species can assemble to a 24-mer upon an increase in divalent cations concentration at neutral pH values [22–24]. In the current research, AfFt was engineered by grafting on it a surface loop of the human H ferritin containing the epitope for the recognition of the human CD71 receptor. The resulting chimeric ferritin (“humanized *Archaeoglobus* ferritin” or HumFt) not only retained its unique assembly properties but was also readily uptaken by different tumor cell lines [25]. In previous work from our research team, this nanoparticle was successfully used to deliver cytochrome c, a pro-apoptotic basic protein, to acute myeloid leukemia cells [26]. This self-assembling nanosystem is particularly suitable for incorporating positively charged molecules due to the anionic nature of the inner surface of the cage. So far, negatively charged molecules, such as nucleic acids, cannot be readily encapsulated within the protein cage. For this reason, we have developed a self-assembling molecular matryoshka in which ferritin traps a positively charged polyamine dendrimer (Poly(amidoamine) or PAMAM) that acts as an anion sponge for negatively charged molecules, nucleic acids in particular. As compared to the plethora of the available cationic vectors, dendrimers stand out because of their unique physical and chemical properties (narrow polydispersity index, nanoscopic size, control over molecular structure) as well as excellent tunable surface characteristics. In addition to the typical electrostatic interactions that drive the binding of nucleic acids, the surface groups of the dendrimer can be directly conjugated with drugs, signal molecules or imaging agents [27–30]. The higher generation dendrimers can also accommodate poorly soluble drugs/bio-actives within their internal structure [31]. The combination of these characteristics made PAMAM dendrimers our cationic vector of choice for the production of an array of hybrid dendrimer-protein nanosystems, particularly for the development of a non-viral vector for the targeted delivery of nucleic acids. The use of PAMAM as a cationic vector for DNA or small RNAs is well documented in the literature [27–30]. PAMAM positive charge typically enables the formation of a strong, yet reversible, complex with negatively charged phosphate backbones of nucleic acids leading to the formation of dendriplexes. However, it is indeed the positive charge that makes PAMAM cytotoxic [32, 33]. The presence of surface cationic charge may cause membrane disruption as well as membrane thinning and erosion, which limits the use of these dendrimers in biological systems. Also, PAMAM dendrimers lack cell specificity unless covalently modified with targeting ligands [34–37]. The hybrid dendrimer-protein nanoparticle we have developed has the purpose to overcome all the limits of non-functionalized PAMAM while preserving its enormous potential.

In the present work, HumFt-PAMAM nanoparticle was tested to deliver a nucleic acid (miRNA-145-5p) to acute promyelocytic leukemia (APL) cells, known to be very difficult to transfect and typically resistant to conventional liposome-based transfection methods. We also evaluated whether this miRNA, upon delivery, could trigger cellular responses.

## Experimental section

### Expression and purification of Humanized Ferritin

*A. fulgidus* humanized ferritin (Hum-Ft) was expressed in *E. coli* and purified (Additional file 1: Figure S1) as previously described [38]. Briefly, 100 g of fermented bacterial paste were resuspended and sonicated in 1 L of 20 mM HEPES buffer pH 7.4 containing 50 mM MgCl_2_ and protease inhibitors. The soluble fraction was treated with 20% (NH_4_)_2_SO_4_. The supernatant was extensively dialyzed versus 10 mM sodium phosphate buffer pH 7.2 containing 20 mM MgCl_2_ and then digested with 20 mg of deoxyribonuclease I (Sigma Aldrich) for 1 h at 37 °C. The sample was centrifuged and subjected to 2 sequential heat treatments at 62 °C and 72 °C. At each step, the denatured proteins were removed by centrifugation, and the soluble fraction was filtered under vacuum using 20 g of Sartoclear Dynamics® Lab Filter Aid (Sartorius) containing highly pure diatomaceous earth (Celpure® C300 – pharmaceutical grade). DNA removal was achieved by a crossflow ultrafiltration step using Vivaflow 200 module (Sartorius) with a cut-off of 100 kD. The same device was used in diafiltration mode to exchange the buffer with 20 mM HEPES pH 7.4 containing 50 mM MgCl_2_.

### PAMAM and HumFt labelling

G4 PAMAM dendrimer (Sigma Aldrich) was labelled with fluorescein-isothiocyanide (FITC) (Sigma Aldrich). 17 μL of PAMAM (57 mM) were added to 2 mL of FITC 1 mg/mL (5.14 mM) in methanol [39]. The reaction was stirred in the dark overnight at room temperature. The PAMAM/FITC solution was dried with a nitrogen stream and resuspended in 2 mL of 20 mM HEPES buffer pH 8.0. The unreacted dye was removed by dialysing the sample versus 20 mM HEPES buffer pH 7.4 for 24 h in the dark. FITC-labelled PAMAM was characterized by HPLC (Additional file 1: Figure S2) and NMR analyses (Additional file 1: Figures S3 and S4).

HPLC analysis was performed with an Agilent Infinity 1260 HPLC apparatus equipped with UV and fluorometric detectors. The separation was carried out using a Halo C18 AQ column (3 × 150 mm, 2.7 µm) connected to the C18 AQ guard column (3 × 5 mm, 2.7 µm). The elution was performed at a flow rate of 0.8 mL/min, with solvent A (0.1% trifluoroacetic acid in water) and solvent B (0.1% trifluoroacetic acid in acetonitrile). The mobile phase was linearly increased from 0 to 100% of solvent B in 15 min and then run isocratically for 5 min. Afterwards, buffer A was reintroduced in the mobile phase up to 100%, and the column was allowed to equilibrate for 10 min. The elution profile of G4 PAMAM was monitored by setting the UV detector at 215 nm. G4 PAMAM/FITC elution was followed by setting the fluorometric detector at λex 490 nm and λem 525 nm.

1H NMR spectra were recorded on a Bruker Avance 400 spectrometer equipped with Prodigy cryoprobe, using D2O/H2O buffer pH 8.5 mixture as solvent at 25 °C. Water signal was reduced by presaturation during relaxation delay and mixing time using the noesygprpr1D sequence (from Bruker suite of programs).

HumFt was labelled with Alexa Fluor 647 NHS ester (succinimidyl ester) (ThermoFisher Scientific) according to the manufacturer’s standard protocol. 10 mg/mL of purified protein were equilibrated in carbonate buffer 0.1 M pH 9.0 containing 100 mM MgCl_2_. 0.1 mL of Alexa Fluor 647 1 mg/mL in DMSO were added to 1 mL of HumFt solution and the reaction mix was incubated for 1 h in the dark at room temperature. To remove the unreacted dye, the labelled protein was dialyzed versus 20 mM HEPES buffer pH 7.4 containing 50 mM MgCl_2_. Protein labelling was checked by UV–vis spectroscopy and High-Performance Size Exclusion Chromatography (HP-SEC) following Alexa Fluor 647 emission signal (λex 650 nm, λem 665 nm) (Additional file 1: Figure S5).

### PAMAM mediated HumFt assembly

G3, G4, G5 PAMAM dendrimers were screened for their ability to induce HumFt association at different HumFt:PAMAM molar ratio (1:10, 1:20, 1:30) in the absence of MgCl_2_. 1 mL of open HumFt 5 mg/mL (10 μM 24-mer) equilibrated in 20 mM HEPES buffer pH 7.4 was added dropwise to 1 mL of dendrimer (100, 200, 300 μM in 20 mM HEPES buffer pH 7.4). Association equilibrium was evaluated by HP-SEC. HP-SEC was performed using an Agilent Infinity 1260 HPLC apparatus equipped with a UV detector. The separation was carried out using an Agilent AdvanceBio SEC 300 Å LC column (7.8 × 150 mm, 2.7 µm). The isocratic analysis was carried out with 20 mM HEPES buffer pH 7.4 as mobile phase. The flow rate was 0.7 mL/min over an elution window of 15 min. HumFt assembly was monitored following UV detection at 220 nm.

### HumFt-PAMAM-miRNA nanoparticle preparation

1 mL open HumFt 5 mg/mL (10 μM 24-mer) equilibrated in 20 mM HEPES buffer pH 7.4 was added dropwise to 1 mL of G4 PAMAM 4.3 mg/mL (300 μM) in the same buffer. MgCl_2_ was added at a final concentration of 50 mM and the sample was incubated overnight at room temperature under stirring. To completely remove free PAMAM, HumFt-PAMAM complex was loaded onto a PD-10 Desalting Column (GE Healthcare) equilibrated with 20 mM HEPES buffer pH 7.4 containing 50 mM MgCl_2_. The protein sample was submitted to a second PD-10 purification step and then concentrated by using Amicon® Ultra-15 Centrifugal Filter Unit with a cut-off of 30 kD to a final protein concentration of 10 μM (Additional file 1: Figure S6).

HumFt-PAMAM nanoparticle stability was tested up to 24 h in (i) 20 mM HEPES buffer containing 50 mM MgCl_2_, (ii) PBS and (iii) RPMI 1640 medium with the addition of penicillin/streptomycin and 10% FCS (Gibco, ThermoFisher Scientific, Waltham, MA, US) by HP-SEC as described above (Additional file 1: Figure S8).

Labelled nanoparticles were prepared in the same condition described above by using Alexa Fluor 647 labelled HumFt and fluorescein labelled G4 PAMAM. Both unlabelled and labelled nanoparticles were analysed by HP-SEC as described above. The isocratic analysis was carried out with 20 mM HEPES buffer pH 7.4 containing 50 mM MgCl_2_ as mobile phase. Nanoparticles containing Alexa Fluor 647 labelled protein or FITC labelled PAMAM were also detected by a fluorometric detector (λex 650 nm and λem 665 nm for Alexa Fluor 647; λex 490 nm and λem 525 nm for FITC).

Protein standard of highly purified HumFt was prepared in the same solution as the mobile phase. HumFt concentration was determined using the theoretical ε_280_ = 32,430 M^−1^ cm^−1^.

An estimate of the number of PAMAM molecules encapsulated into the ferritin shell was performed by HPLC using a Halo C18 AQ column, following the fluorescence signal of PAMAM/FITC as described above. Highly purified HumFt-PAMAM/FITC complex was disassembled in the absence of magnesium ions and in the presence of 20% methanol. After centrifugation, the supernatant was directly analyzed by HPLC. Quantitative results were obtained using standard curves for G4 PAMAM and G4 PAMAM/FITC (considering 3.3 FITC molecules bound to each PAMAM according to ^1^HNMR experiments, Additional file 1: Figure S4).

HumFt-PAMAM-miRNA and HumFt/FITC-PAMAM-miRNA nanoparticles were prepared adding pre-miRNA-145 (miR-145-5p precursor #AM17100, assay ID PM11480, ThermoFisher Scientific, Waltham, MA USA) or pre-miRNA Precursor Negative Control (#AM17110, ThermoFisher Scientific, Waltham, MA, USA), at a final concentration of 133 nM, to the preassembled HumFt-PAMAM nanoparticle (10 μM) and incubating at 4 °C for 5 h. The formation of the ternary complex with miRNA was monitored by HP-SEC following the absorbance at 260 nm and agarose gel electrophoresis coupled to a GelDoc Imaging System (Biorad). All solutions were prepared by using RNase-free water, tips, and tubes.

### Negative staining electron microscopy

Formvar-carbon coated grids were floated onto 20 μL of protein solutions (0.02 mg/mL) for 5 min for adsorption and the excess of sample solution was blotted gently by filter paper. The air-dried grids were stained with an aqueous solution of 2% (w/v) uranyl acetate or 4% ammonium molybdate for 30 s. The excess staining solution was removed carefully. The grids were observed with an EM208S transmission electron microscope (FEI—ThermoFisher Scientific; 208 Eindhoven—The Netherlands) at an acceleration voltage of 100 kV. Electron micrographs were taken with a slow-scan camera (MEGAVIEW III, OLYMPUS).

High magnification protein images were analysed by the freeware software ImageJ (version 1.29, NIH, Bethesda, MD). To calculate the diameter size of the proteins, manual measures of more than 150 particles were taken for each sample and the diameter size distributions were conducted by Excel 2016.

### Cell culture, cell death, and differentiation

The NB4 cell line was obtained by DSMZ (Branuschweig, Germany). Cell cultures were routinely tested for mycoplasma contamination and the cells were cultured and treated in RPMI 1640 medium with the addition of penicillin/streptomycin and 10% FCS (Gibco, ThermoFisher Scientific, Waltham, MA, US). NB4 cells were treated with 0.3 μM HumFt, HumFt-PAMAM, HumFt/FITC-PAMAM-pre-miRNA, and HumFt-PAMAM-pre-miRNA nanoparticles prepared as described above.

Cell density was measured by trypan blue exclusion assay using a Burker chamber. Cell death was analyzed by flow cytometry (CytoFLEX, Beckman Coulter, Brea, CA, US) after staining the cells with 2.5 μg/mL propidium iodide (Sigma Aldrich). Cell differentiation was assessed by morphological analysis of cytospin preparations, obtained by spinning about 300,000 cells/slide, stained with Wright-Giemsa (Sigma Aldrich). Slides were examined with a Zeiss Axioskop 2 microscope and images were acquired with the AxioCam HRc and the Axiovision 4.8 software (Zeiss, Oberkochen, Germany). The nucleus/cytosol ratio was calculated after measuring the area of nucleus and cytosol of at least 60 cells in two different fields for each sample, by the Image J/Fiji software (https://imagej.nih.gov). Statistical analysis was performed by one-way ANOVA with Prism software.

### RNA extraction and Real-Time PCR

48 h after addition of HumFt-PAMAM nanoparticle or HumFt-PAMAM-pre-miRNA nanoparticles, cells were counted and 10^6^ cells for each sample were used to extract total RNA. Total RNA was isolated by TRIzol (Ambion, ThermoFisher Scientific, Waltham, MA, USA) following the manufacturer’s instructions and the quality of purified RNA was checked by OD 260–280 readings. 250 ng of total RNA was reverse-transcribed with the High-Capacity RNA to cDNA kit (Applied Biosystems, ThermoFisher Scientific, Waltham, MA, US) in a final volume of 8 μL. cDNA was diluted in RNase-free water and 1 μL of this was used for Real-Time PCR to assess the expression of RARα gene by TaqMan MicroRNA assay (Applied Biosystems, Foster City, CA, US) following the manufacturer’s instructions. The fold increase of RARα was calculated by the ΔΔCt method using the GAPDH gene as the endogenous control for standardization. Reverse transcription of total RNA for detection of miR-145-5p was performed using miScript II RT kit (Qiagen, Chatsworth, CA). Quantification of mature miR-145-5p expression was carried out by miScript SYBR Green PCR kit (Qiagen, Chatsworth, CA, US), using miScript Primer Assay Hs_miR-145_1 (MS00003528 Qiagen, Chatsworth, CA). The fold increase of miRNA-145-5p was calculated by the ΔΔCt method using Hs_SNORD68 RNA (miScript Primer Assay MS00033740 Qiagen, Chatsworth, CA) as the endogenous control for standardization. All reactions were performed in the Quant Studio 7 Real-Time PCR system (Applied Biosystems, ThermoFisher Scientific, Waltham, MA, US). The experiment was performed in biological duplicates and one-way ANOVA statistical analysis was performed using the GraphPad-Prism 6 software (GraphPad Software, La Jolla, CA, US).

### Confocal microscopy and FACS analysis

NB4 cells were incubated for 24 h or 48 h with 0.3 μM G4 PAMAM/FITC, or with 0.3 μM HumFt-Alexa Fluor 647, or with 0.3 μM of the double dye-labelled HumFt-PAMAM nanoparticle, or with 0.3 μM HumFt/FITC-PAMAM-pre-miRNA nanoparticle prepared as described above. The uptake of the labelled molecules was assessed by flow cytometry (CytoFLEX, Beckman Coulter, Brea, CA, US) and the data were analyzed by the CytExpert Software (Beckman Coulter, Brea, CA, US).

The same cells were examined by confocal microscopy to assess the intracellular localization of the compounds. Cytospin preparations obtained as described above were fixed in 4% PFA for 7 min. DNA was counterstained with Hoechst 33,342. Confocal laser scanning microscopy (CLSM) was performed using a Zeiss LSM 980 with Airyscan2, using the 63 × oil objective and excitation spectral laser lines at 405, 488, and 633 nm. Signals from the different fluorescent probes (Hoechst, FITC, and Alexa Fluor 647) were taken in sequential scanning mode to avoid spectral overlap (false-positive signal). CLSM analysis was performed both at the central optical section of each field of observation and as a 3d reconstruction of z-stacks. Co-localization areas were detected in orange. Bright-field images were taken to identify cell morphology. Scale bars are 5 µm.

## Results and discussion

### PAMAM mediated HumFt assembly

**Fig. 1:**
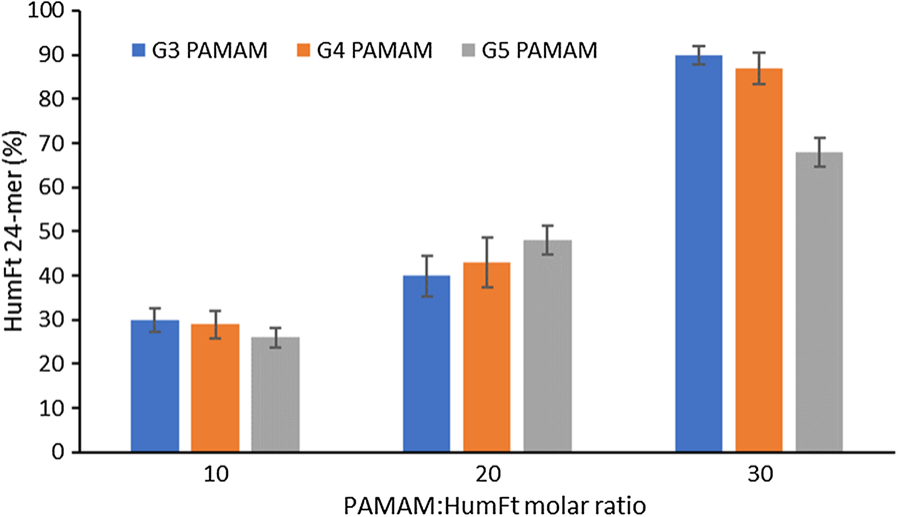
PAMAM-induced HumFt assembly. Ferritin assembly (24-mer %) was evaluated in the absence of magnesium ions adding 10, 20 and 30 molar excess of different generations of PAMAM (G3, G4 and G5) (mean ± SD, n = 3)

HumFt is endowed with unique assembly-disassembly properties that promote divalent cation-dependent polymerization into a 24-meric associated state. So far, HumFt is potentially able to accommodate relatively large molecules with a diameter up to 6–7 nm, provided that they display an overall positive charge. The PAMAM dendrimers tested in the present work fulfil such requirements, in terms of charge and size, and are shown to be readily embedded into the protein shell. In the first step we tested 3 different PAMAM generations (G3, G4, G5) and we evaluated whether they were able, thanks to their highly positive charge, to mediate protein assembly even in the absence of magnesium ions. These PAMAM generations were selected in such a way that their diameters (34, 45, 54 Å) could fit the internal cavity of the ferritin cage (90 Å). As shown in Fig. 1, all tested PAMAMs were able to mediate ferritin reassembly in a dose-dependent manner.

At least 30 molar excesses of G3 and G4 PAMAMs were required to obtain full closure of the protein cage (comparable to that obtained with magnesium ions). On the other hand, G5 PAMAM was less efficient in mediating protein closure. Given the reported optimal nucleic acid binding properties of G4 PAMAM, it has been selected as the candidate of choice for the development of this hybrid nanoparticle. The size-to-charge ratio is crucial to obtain stable and uniform RNA-dendrimer complexes and G4 PAMAM is the lowest threshold dendrimer generation for effective small RNA complexation [30, 40, 41].

**Fig. 2:**
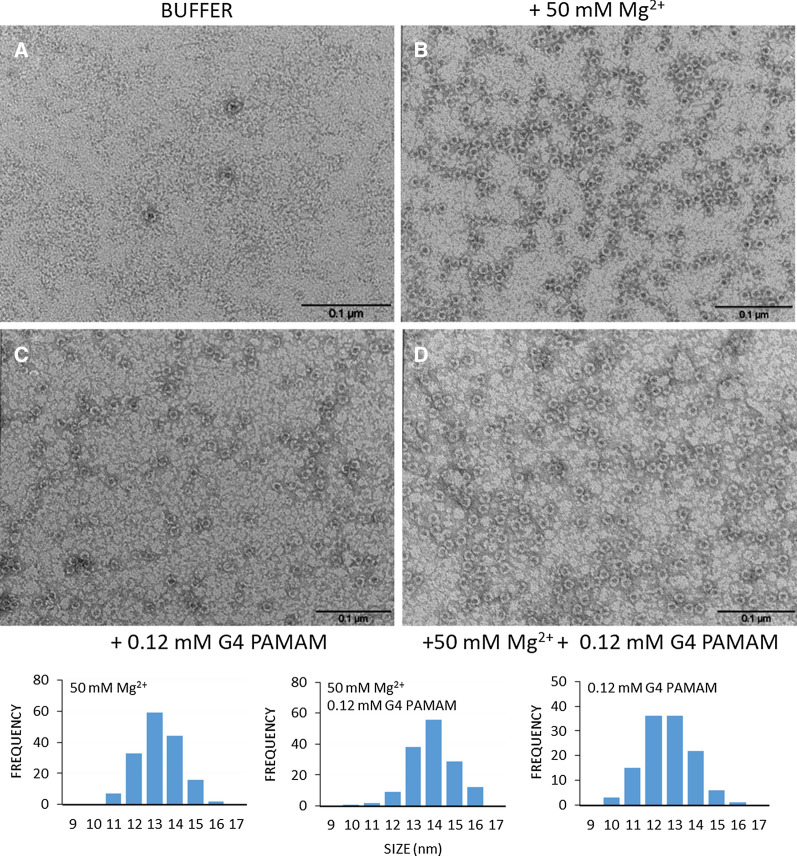
TEM analysis of HumFt. **A** HumFt in HEPES buffer without magnesium ions; **B** 50 mM Mg^2+^ and/or **C** and **D** G4 PAMAM mediate protein nanoparticle assembly. Scale bar: 0.1 μm

To confirm the correct ferritin assembly in the presence of G4 PAMAM, TEM analyses were carried out. The acquisitions were performed using 2% uranyl acetate (pH 4.5) or 4% ammonium molybdate as the contrast agent. In all samples, the electron dye interacts with the protein 24-mer resulting in a characteristic ring shape typical of correctly assembled ferritins. As expected, the absence of Mg^2+^ shifts the equilibrium towards the open dimeric form (Fig. 2A), while the addition of divalent cations and/or G4 PAMAM shifts the equilibrium towards the closed form (Fig. 2B–D). The measurements confirm a homogeneous protein population in the expected shape and size (about 13 nm).

**Fig. 3:**
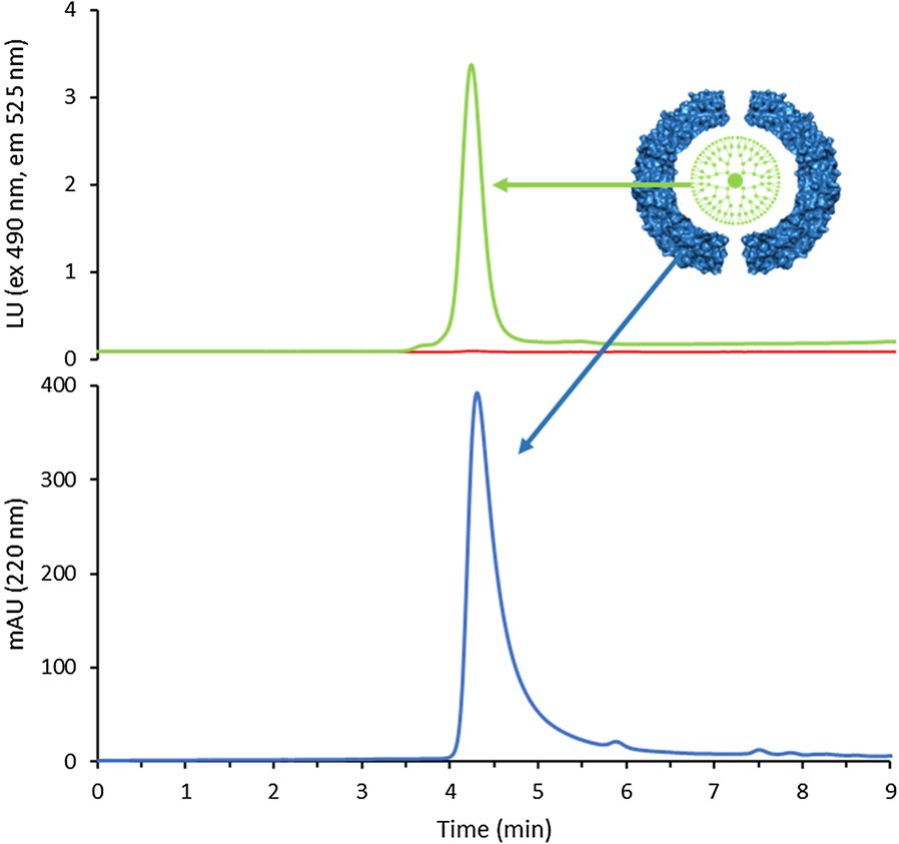
HP-SEC analysis of HumFt-PAMAM nanoparticle. G4 PAMAM was labelled with FITC and encapsulated into HumFt protein shell. The hybrid nanoparticle formation was monitored following the fluorescence signal (green line) of G4 PAMAM/FITC (ex 490 nm, em 525 m) at the retention time (4.5 min) of ferritin 24-mer (UV chromatogram, blue line). Empty ferritin does not show intrinsic fluorescence upon excitation at 490 nm (red line)

The HP-SEC measurements were performed to confirm that PAMAM actually forms a complex with ferritin (Fig. 3). In particular, FITC labelled PAMAM encapsulation was monitored by following the fluorescence signal at the retention time of the closed ferritin (4.5 min). Acquisitions were carried out by following the UV (220 nm) and fluorescence (λex 490 nm, λem 525 nm) signals simultaneously. Since HumFt does not exhibit an intrinsic fluorescence under these analytical conditions, the peak at 4.5 min in the fluorescence chromatogram (upper panel) could only be explained by the presence of PAMAM/FITC in its internal cavity. In addition, the UV absorption chromatogram (lower panel) indicates that HumFt was almost completely in its closed form (the dimeric form eluting at 6 min is less than 2%). These data suggest a synergistic action of PAMAM and magnesium because, in the presence of divalent cations only, dimeric ferritin is about 10–15% (Additional file 1: Figure S7).

An estimate of the number of G4 PAMAM molecules encapsulated into the ferritin shell was performed by HPLC, after disassembling the highly purified HumFt-PAMAM/FITC complex. The results obtained show that an average of 1.1 G4 PAMAM molecules per HumFt 24-mer. This result is in agreement with the dimensions of both the PAMAM G4 (4.5 nm) and the protein cavity (9 nm). It is very unlikely that 2 molecules of PAMAM could coexist in the reduced space of the protein cavity, considering their electrostatic repulsions.

HumFt-PAMAM nanoparticle stability was tested up to 24 h at 37 °C in i) 20 mM HEPES buffer containing 50 mM MgCl_2_, ii) PBS buffer, and iii) RPMI 1640 medium containing 10% FCS. In all the tested condition the hybrid nanoparticle is stable, being the HumFt 24-mer always higher than 95% (Additional file 1: Figure S8).

### Uptake of HumFt-PAMAM nanoparticles by NB4 cells

Once the ability of HumFt to encapsulate G4 PAMAM was established, the internalization of this new hybrid nanoparticle into cells was tested. For the uptake experiments, we selected an acute promyelocytic leukemia cell line (NB4), which is typically hard to transfect and is resistant to conventional liposome-based transfection methods. This cell line has already been successfully tested in a previous study in which HumFt delivered a therapeutic protein [26].

**Fig. 4:**
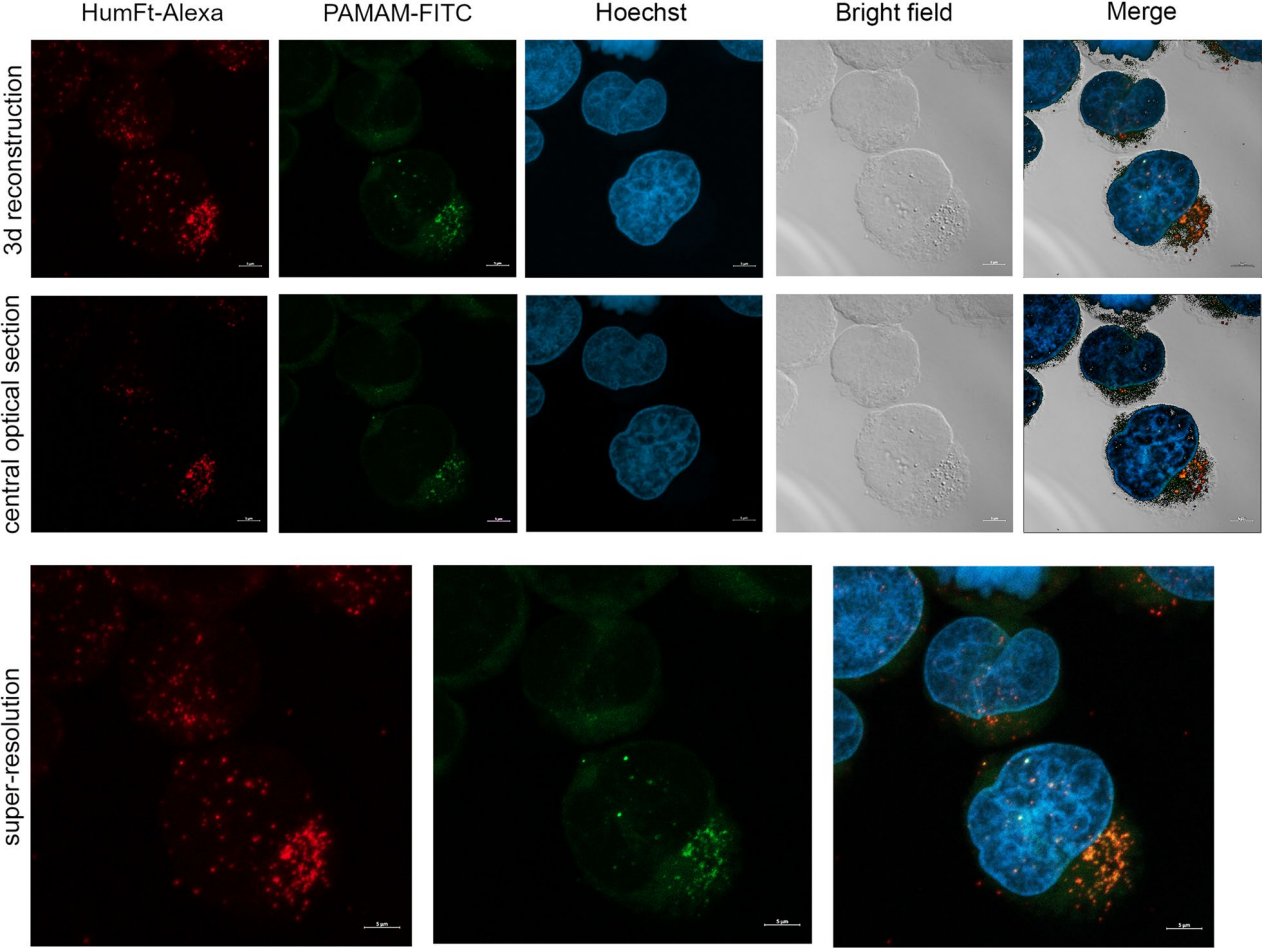
Confocal microscopy of APL NB4 cells treated with double dye-labelled HumFt-PAMAM nanoparticles. HumFt (red) and G4 PAMAM (green) colocalized in the cell cytosol. Co-localization areas were detected in orange. The nuclei were stained with Hoechst 33,342 (blue). Scale bar: 5 μm

A differential labelling was used to evaluate the uptake of the HumFt-PAMAM nanoparticle by cells: G4 PAMAM was FITC labelled (green) (Additional file 1: Figures S2–S4), whereas HumFt was marked with Alexa Fluor 647 (red) (Additional file 1: Figure S5). The correct protein labelling was verified both by UV–vis spectroscopy and by HP-SEC analysis by following the signal of the Alexa Fluor 647 (λex 650, λem 665). Attempts to label the protein as a dimer appeared to impair the nanoparticle's ability to assemble properly (Additional file 1: Figure S5). This is probably due to the modification of lysine residues involved in the self-assembly process of the 24-meric polymer. Double dye-labelled HumFt-PAMAM nanoparticle was then used to evaluate the uptake by NB4 cells by FACS (Additional file 1: Figure S9) and confocal microscopy (Fig. 4). Confocal analysis showed that the hybrid nanoparticle is uptaken by cells. As shown in Fig. 4, HumFt (red) and PAMAM (green) co-localize (orange) confirming that the dendrimer is indeed conveyed by the protein shell. The 3-D reconstruction, as well as the central optical section, showed that the hybrid nanoparticles were located exclusively in the cytoplasm.

### HumFt-PAMAM mediated miRNA delivery to NB4 cells

**Fig. 5:**
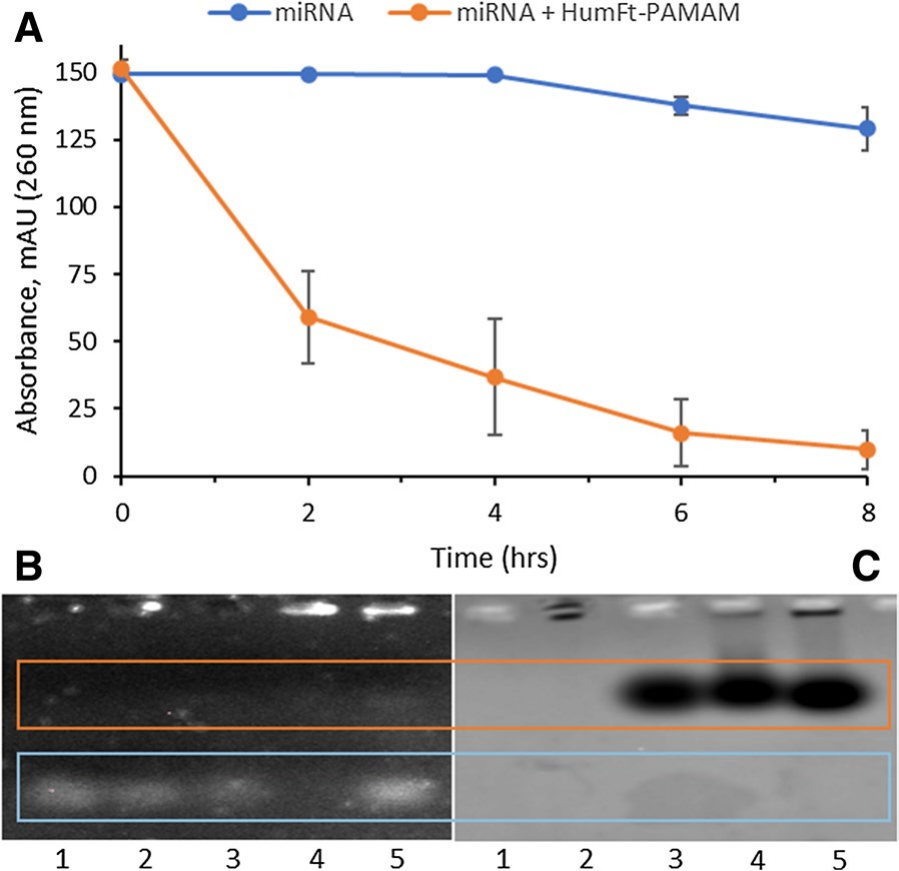
Formation of the ternary complex HumFt-PAMAM-miRNA. **A** HP-SEC analysis of miRNA with (orange line) and without (blue line) HumFt-PAMAM, followed at 260 nm. **B** Agarose gel electrophoresis stained with SYBR Safe and **C** agarose gel electrophoresis stained with Coomassie Blue. Lane 1: miRNA; lane2: PAMAM-miRNA; lane 3: HumFt-miRNA; lane 4: HumFt-PAMAM-miRNA; lane 5: HumFt-PAMAM-miRNA at 99 °C

HumFt-PAMAM hybrid nanoparticle was designed to deliver small RNAs to target cells. The advantage of this system over previously published ones [29] consists of the simplicity and speed of assembly, which does not require covalent modifications of any kind. This system is self-assembling as it is sufficient to add PAMAM and magnesium ions to the dimeric ferritin to favour the closure of the protein cage which, as expected, draws inside the small nucleic acid. Under the current experimental set-up, a pre-miRNA was used, that enters the protein cavity through the 4 large triangular openings, attracted by the positive charge of PAMAM. The formation of the ternary complex HumFt-PAMAM-pre-miRNA was monitored by HP-SEC and electrophoretic mobility shift assay (EMSA). As shown in Fig. 5A, when miRNA is incubated with HumFt-PAMAM nanoparticle, free miRNA signal decreases over time and after 8 h its concentration in less than 10% (< 10 nM) (orange line). In the absence of HumFt-PAMAM nanoparticle miRNA concentration is almost constant over time (blue line). The same results were obtained by EMSA analysis. As shown in Fig. 5B, neither ferritin, nor free PAMAM determine an electrophoretic shift compared to the reference miRNA (lanes 1–3, blue box). When miRNA is incubated with the hybrid nanoparticle, the unbound miRNA signal disappears (lane 4), indicating the formation of the ternary complex. The lack of the signal could be explained by a shielding effect of the nanoparticle. After heat treatment (99 °C), the ferritin nanocage loses its quaternary structure releasing the oligonucleotide (lane 5). Staining of the same gel with Coomassie blue (Fig. 5C) shows the position of the protein nanoparticle. Together these data demonstrate the presence of the nucleic acid within the HumFt-PAMAM nanoparticle.

**Fig. 6:**
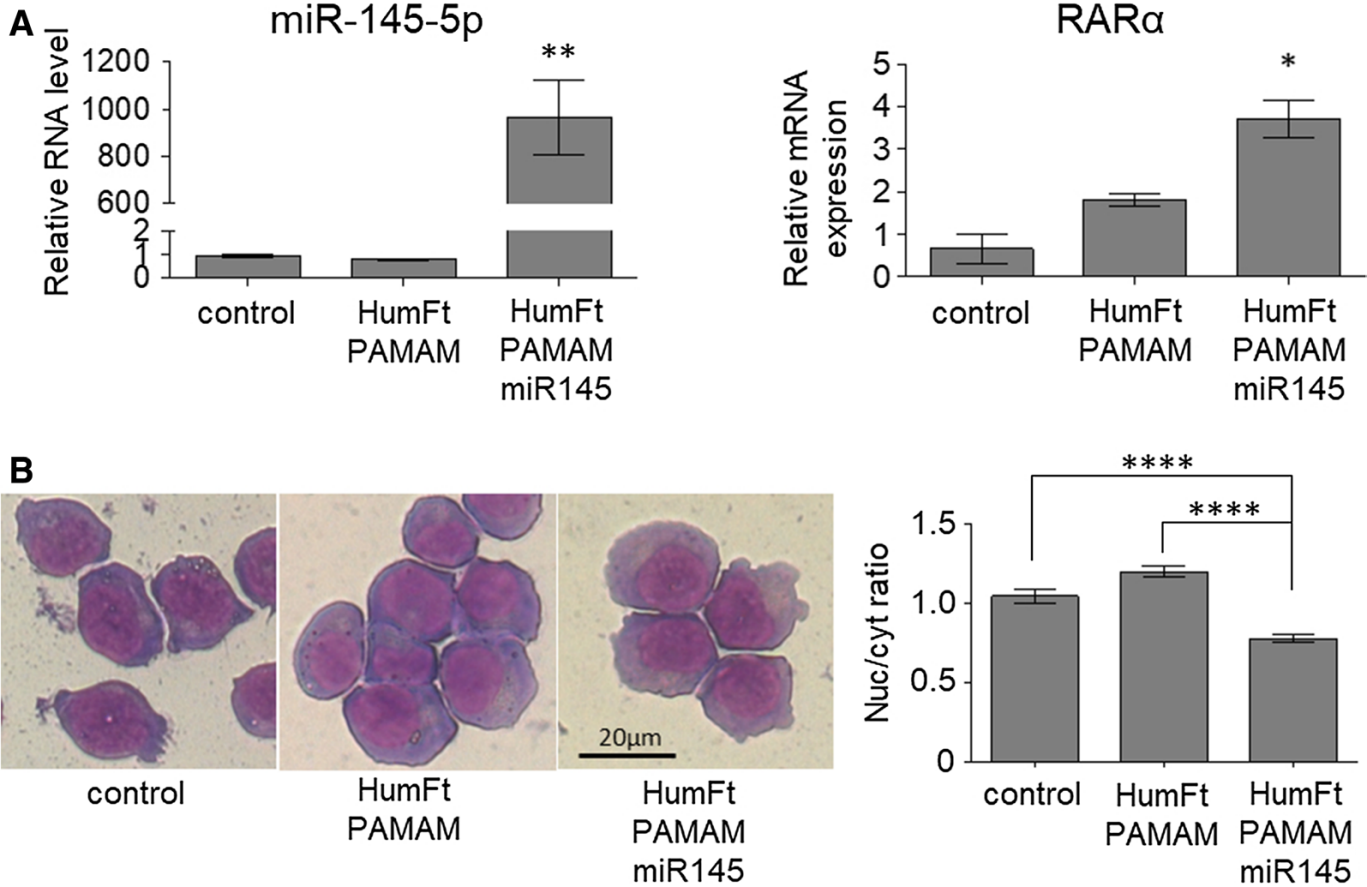
miRNA-145-5p delivery by the HumFt-PAMAM nanoparticle promotes differentiation of the NB4 cell line. **A** NB4 cells were incubated with the HumFt-PAMAM-pre-miRNA-145-5p nanoparticle for 48 h. qRT-PCR revealed high levels of miRNA-145-5p (p < 0.01) and increased expression of RARα mRNA. (mean ± SEM, n = 2, p < 0.05, one-way ANOVA statistical analysis relatively to control). **B** Morphological analysis of NB4 cells treated as in (**A**) evidenced that miRNA-145-5p promotes differentiation, as indicated by cytosol enlargement, Golgi expansion, and decreased basophilia compared to control cells or to cells treated with the HumFt-PAMAM nanoparticle without pre-miRNA-145-5p (left panel). The right panel reports the ratio between the area of nucleus and cytosol (n ≥ 60 ± SEM cells from at least two different fields; statistical analysis one way ANOVA)

The ternary nanoparticle was used to deliver pre-miRNA to NB4 cells. At present, the only successful transfection methods for these cells were electroporation or, alternatively, the use of lentiviral vectors. Electroporation is an efficient method for transient or stable nucleic acid delivery in immune cells, but the main drawback is a high rate of cell death [42]. This can be particularly troublesome for NB4 cells, which grow in suspension. On the other hand, lentiviral transduction is an efficient method for stable DNA delivery in hard to transfect cells [43], but it requires proper equipment and biosafety level 2 cell culture rooms. Furthermore, since the exogenous DNA is integrated into the host cell genome, this method also displays risks related to mutagenesis/tumorigenesis potential [44, 45]. The development of the present hybrid nanoparticle fits into this frame and has the purpose to overcome the drawbacks of classical transfection methods by providing an efficient and non-toxic system for cell cultures. To set up this delivery system, we have chosen as a proof of concept a microRNA, as it can be easily detected by Real-Time PCR once delivered to and released into NB4 cells. In particular, we have chosen miRNA-145-5p because, being involved in granulocytic differentiation of APL cells, it allows the detection of possible effects on cell phenotype upon its delivery [46]. In fact, NB4 cells are arrested by the oncogenic driver fusion protein PML-RARα at the promyelocytic stage of the differentiation process that leads hematopoietic progenitor cells to mature granulocytes. This oncogenic differentiation block can be totally or partially removed by appropriate stimuli like pharmacological amounts of retinoic acid, forced down-regulation, or over-expression of genes and non-coding RNA involved in the differentiation process [47, 48]. Upon incubation with the HumFt-PAMAM-pre-miR145-5p nanoparticle, NB4 cells maintained the same proliferation and viability rates (Additional file 1: Figure S11) and displayed very high levels of miR145-5p. Specific detection of mature miRNA-145-5p by qRT-PCR indicates that the miRNA precursor loaded in the HumFt-PAMAM nanoparticle was released in the cytosol and properly processed by Dicer RNase. The presence of miR-145-5p correlated with significantly increased expression of the Retinoic Acid Receptor α (RARα), an early hallmark of granulocytic differentiation (Fig. 6A). Accordingly, cell morphology analysis showed signs of differentiation in the cells incubated with the ternary nanoparticle as suggested by reduced nucleus/cytosol ratio, decreased basophilia, and enlarged Golgi (Fig. 6B and Additional file 1: Figure S10). qRT-PCR analysis of late differentiation markers showed that miRNA-145p overexpression was not sufficient to achieve full differentiation (data not shown). Nonetheless this is not surprising in the absence of the differentiation stimulus of retinoic acid. Hence, such a result indicated that the nanoparticle was able to efficiently transport the nucleic acid inside the cell, which escapes the endosome leading to pre-miRNA processing and inducing the observed phenotypic effects. Nevertheless, the precise molecular mechanisms underlying ferritin cellular trafficking are not yet fully understood [49]. HumFt was engineered to recognize the CD71 receptor, which is physiologically used by both transferrin and serum ferritin. Typically, transferrin, once endocytosed, is recycled by exocytosis, while ferritin is released into the cytosol, where it functions as an iron-storage protein. Cytosolic release most likely occurs through an endosomal escape process, not yet fully described and characterized. To the best of our knowledge, ferritin typically localizes in the early endosomes and is not sent to the Golgi apparatus or lysosomes by late endosomes [50]. As endocytosed ferritin is not recognized by the cell as something to degrade, it can be considered an ideal nanosystem for drug delivery. Our ferritin carries the PAMAM molecule, which may still contribute to the endosomal escape of the hybrid nanoparticle. When PAMAM is used as a non-viral vector, it is internalized into the endosomes and the unprotonated ternary amines within its branched structure buffer the acidic pH of these vesicles [51, 52]. This results in more protons being pumped in, with a concomitant chloride accumulation, which enhances the nucleic acid transfer by dendriplexes [53]. The osmotic swelling enhances the local membrane tension and the direct interaction with the positively charged PAMAM causes the rupture of the endosomal membrane, eventually leading to the release of its contents into the cytoplasm [54, 55]. However, in the hybrid nanosystem we have developed, the PAMAM is protected within the ferritin cavity, and this prevents the direct interaction of the dendrimer with the membrane, which is typically crucial for its rupture. Nevertheless, our data show that endosomal escape occurs also in the case of our hybrid nanoparticle (regardless of the buffering effect of the dendrimer [26]), which localizes into the cytosol, as clearly demonstrated by confocal microscopy (Fig. 4). In addition, the nucleic acid release is confirmed by the pre-miRNA processing to mature miRNA by Dicer nuclease, which typically occurs in the cytoplasm. At present we are not able to establish whether the endosomal escape of the hybrid nanoparticle follows the intracellular ferritin pathway and to what extent it could also be mediated by the proton-sponge effect of PAMAM.

Escape from endosomes is always a key rate-limiting step in small RNA efficiency [56], which can be improved only by using a highly efficient delivery system. From this point of view HumFt is the ideal candidate because, as demonstrated by the FACS data, 100% of the cells are loaded with HumFt (Additional file 1: Figure S9 and Figure S11), thus increasing the possibility that the payload is uptaken as well.

## Conclusions

In the present work, we have shown that the HumFt-PAMAM hybrid nanoparticle is a transfection system capable of delivering miRNAs into cells expressing CD71 receptors. Once internalized, pre-miRNA is released into the cytosol and processed by Dicer nuclease to mature miRNA, thus eliciting phenotypic effects in NB4 leukemia cells similar to early-stage differentiation. In particular, NB4 cells showed enhanced expression of RARα, as well as morphological changes typical of the granulocyte differentiation process, upon miRNA-containing nanoparticle uptake. The great potential of this system is exploited by the unique features of its constituents: (i) PAMAM dendrimers are typically very versatile as they naturally interact with small nucleic acids regardless of their sequence; (ii) HumFt confers selectivity towards CD71 overexpressing cells, thus limiting the potential cytotoxicity and uncontrolled uptake of free PAMAM and protecting the nucleic acid from degradation. The self-assembly strategy here presented not only yields homogeneous hybrid nanoparticles in the expected shape and size but also provides an efficient tool for non-covalent functionalization of nanoparticles with an extraordinary high range of affinity molecules. This approach provides a future blueprint for the design of a new family of transfecting agents that can specifically target a wide range of diseased cells.

## Founding

This research was funded by Sapienza University of Rome, “Progetto Ateneo 2019” to Alberto Boffi and by AIRC IG 2018—ID. 21406 project, Istituto Pasteur Italia—Fondazione Cenci Bolognetti to Francesco Fazi.

## Supplementary Information

**Additional file 1: Figure S1. **HumFt purification. **Figure S2.** HPLC analysis of purified G4 PAMAM-FITC. **Figure S3.** 1HNMR analysis of G4 PAMAM-FITC. **Figure S4.** Estimation of the FITC/G4 PAMAM ratio. **Figure S5.** UV-vis and HP-SEC analysis of Alexa Fuor 647 labelled HumFt. **Figure S6.** HumFt-PAMAM nanoparticle synthesis: free G4 PAMAM removal by PD10 column. **Figure S7.** G4 PAMAM shifts the equilibrium towards closed HumFt (24-mer). **Figure S8.** HumFt-G4 PAMAM nanoparticle stability. **Figure S9.** FACS analysis of NB4 cells treated with HumFt-PAMAM hybrid nanoparticles. **Figure S10.** Proliferation and viability of NB4 cells treated with HumFt-PAMAM-miR145 hybrid nanoparticle. **Figure S11.** Morphological analysis of NB4 cells treated with HumFt-PAMAM-miR145 nanoparticle.

## Data Availability

All data related to the manuscript are available in the manuscript and in the Supplementary Information in the form graphs and figures.
