# Supplementary material for: Self-assembling ferritin-dendrimer nanoparticles for targeted delivery of nucleic acids to myeloid leukemia cells
Source: J Nanobiotechnology. 2021 Jun 9;19:172. doi: 10.1186/s12951-021-00921-5 (PMC8190868; doi:10.1186/s12951-021-00921-5)
Supplement: Supplementary file 1 — Additional file 1: Figure S1. HumFt purification. Figure S2. HPLC analysis of purified G4 PAMAM-FITC. Figure S3. 1HNMR analysis of G4 PAMAM-FITC. Figure S4. Estimation of the FITC/G4 PAMAM ratio. Figure S5. UV-vis and HP-SEC analysis of Alexa Fuor 647 labelled HumFt. Figure S6. HumFt-PAMAM nanoparticle synthesis: free G4 PAMAM removal by PD10 column. Figure S7. G4 PAMAM shifts the equilibrium towards closed HumFt (24-mer). Figure S8. HumFt-G4 PAMAM nanoparticle stability. Figure S9. FACS analysis of NB4 cells treated with HumFt-PAMAM hybrid nanoparticles. Figure S10. Proliferation and viability of NB4 cells treated with HumFt-PAMAM-miR145 hybrid nanoparticle. Figure S11. Morphological analysis of NB4 cells treated with HumFt-PAMAM-miR145 nanoparticle. [file 12951_2021_921_MOESM1_ESM.docx]

**SUPPLEMENTARY INFORMATION**

**Self-assembling ferritin-dendrimer nanoparticles for targeted delivery of nucleic acids to myeloid leukemia cells**

**Federica Palombarini*^1^, Silvia Masciarelli*^2,4^, Alessio Incocciati^1^, Francesca Liccardo^2^, Elisa Di Fabio^1^, Antonia Iazzetti^3^, Giancarlo Fabrizi^3^, Francesco Fazi^#2^, Alberto Macone^#1^, Alessandra Bonamore^#1^, Alberto Boffi^1,5^**

**^1^** Department of Biochemical Sciences "Alessandro Rossi Fanelli", Sapienza University of Rome, Piazzale Aldo Moro 5, 00185 Rome Italy.

^2^ Department of Anatomical, Histological, Forensic & Orthopaedic Sciences, Section of Histology and Medical Embryology, Sapienza University of Rome, Via A. Scarpa, 14-16, 00161 Rome, Italy; laboratory affiliated to Istituto Pasteur Italia-Fondazione Cenci Bolognetti.

^3^ Department of Chemistry and Technology of Drugs, Sapienza University of Rome, Piazzale Aldo Moro 5, 00185 Rome Italy.

^4^Histology and Embryology section, Department of Life Science and Public Health, Fondazione Policlinico Universitario A. Gemelli IRCCS, Largo Agostino Gemelli 8, 00168 Roma, Italy.

^5^Center for Life Nano Science@Sapienza, Istituto Italiano di Tecnologia, V.le Regina Elena 291, Rome 00161, Italy.

**Figure S1: HumFt purification**

**Figure S2: HPLC analysis of purified G4 PAMAM-FITC**

**Figure S3: ^1^HNMR analysis of G4 PAMAM-FITC**

**Figure S4: estimation of the FITC/G4 PAMAM ratio**

**Figure S5: UV-vis and HP-SEC analysis of Alexa Fuor 647 labelled HumFt**

**Figure S6: HumFt-PAMAM nanoparticle synthesis: free G4 PAMAM removal by PD10 column**

**Figure S7: G4 PAMAM shifts the equilibrium towards closed HumFt (24-mer)**

**Figure S8: HumFt-G4 PAMAM nanoparticle stability**

**Figure S9:** **FACS analysis of NB4 cells treated with HumFt-PAMAM hybrid nanoparticles**

**Figure S10: Proliferation and viability of NB4 cells treated with HumFt-PAMAM-miR145 hybrid nanoparticle**

**Figure S11: Morphological analysis of NB4 cells treated with HumFt-PAMAM-miR145 nanoparticle**

**Figure S1: HumFt purification**

HumFt was purified using a chromatography free purification protocol based on heat treatments and crossflow ultrafiltration. Protein purity was ≥ 99% as checked by SDS-PAGE and HP-SEC analysis. In the denaturing conditions of SDS-PAGE HumFt runs as a monomer of about 19 kD.

HP-SEC chromatogram of purified ferritin was performed in the presence of MgCl_2_. The chromatogram shows two main peaks relative to the 24-meric and dimeric protein. The equilibrium is neatly shifted towards the 24-meric form.

**Figure S2: HPLC analysis of purified G4 PAMAM-FITC**

G4 PAMAM was labelled with fluoresceine isothiocyanate (FITC), extensively dialyzed, and analyzed by HPLC using a C18 Halo column. G4 PAMAM (red line, retention time 4.8 min) was quantitatively converted in its FITC-derivative (green line, retention time 9.6 min). Unlike unlabelled G4 PAMAM, the FITC-derivative shows a fluorescence signal upon excitation at 490 nm (upper chromatogram). Free FITC elutes at 10.7 min (blue line) and is not detectable in G4 PAMAM-FITC sample (green line), indicating that extensive dialysis completely removes it.

**Figure S3: ^1^HNMR analysis of G4 PAMAM-FITC**

G4 PAMAM dendrimer was conjugated with FITC through the thiourea linkage. G4 PAMAM derivatization was confirmed by ^1^H NMR analysis in D_2_O at pH 8.5. ^1^H NMR spectra of G4 PAMAM-FITC adduct are recorded in D_2_O/H_2_O buffer pH 8.5 mixture at 25°C.

The ^1^H NMR spectrum of G4 PAMAM-FITC adduct clearly shows the FITC presence in the adduct due to the signals of the proton 1-6 of the tricyclic core.


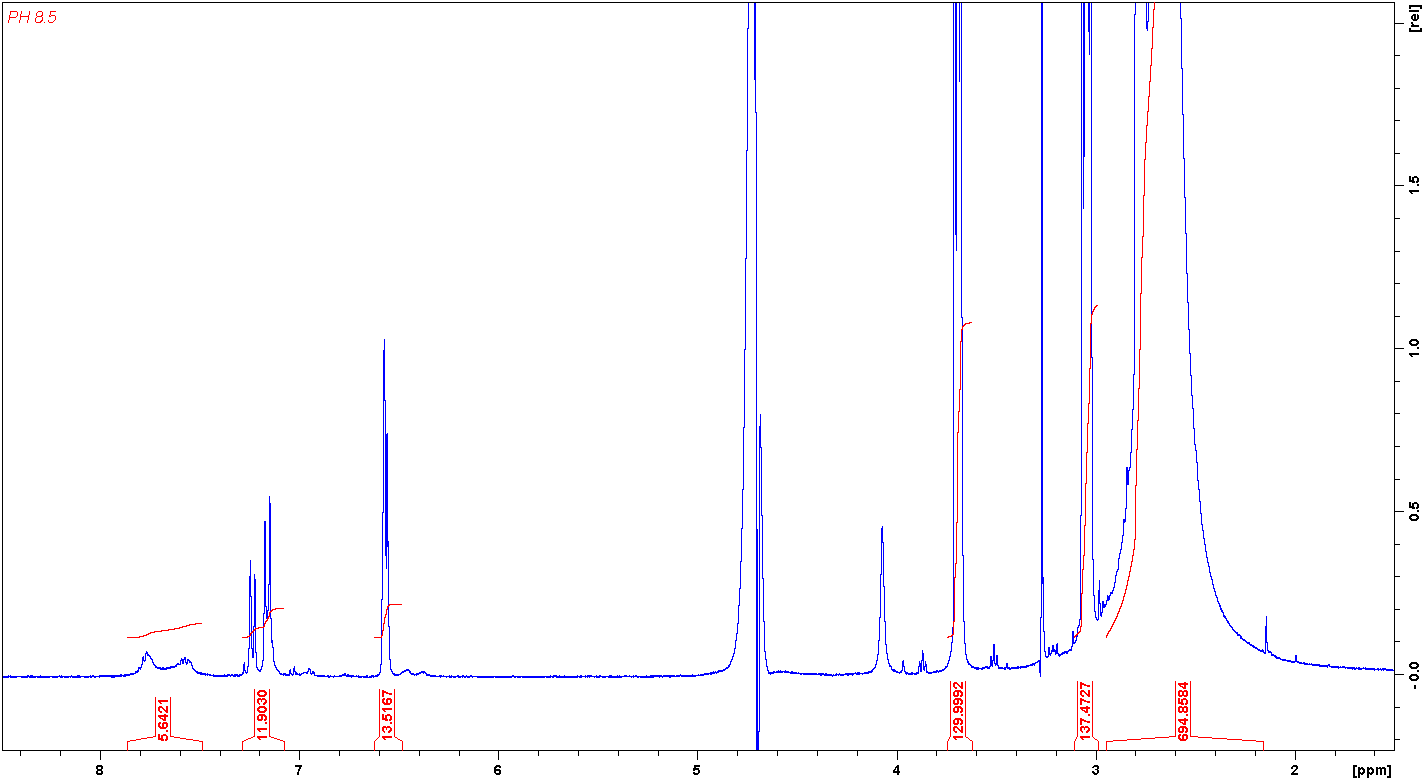


H^5^ + H^5^ (2H)

H^1^ + H^2^ (4H)

H^3^ + H^4^ (3H)

**Figure S4: estimation of the FITC/G4 PAMAM ratio**

^1^HNMR superimposition spectra of G4 PAMAM-FITC (blue line), and pure FITC (red line in D_2_O/H_2_O buffer pH 8.5 mixture at 25°C. As regards the estimation of the FITC/G4 PAMAM ratio, we proceeded as follows: the area of ​​all methylene groups of the dendrimer (3.75 to 2.00 ppm) was normalized to 996 H, corresponding to 498 methylene units; subsequently, the triplet area at 3.69 ppm, J = 5.82 Hz most likely the -CONHCH2 group of the dendrimer, becomes 130 H, in excellent agreement with the theoretical value of 128 (64 equivalent methylene groups). Consequently, the value of 13.5 of the integral of the multiple from 6.60 to 5.53 (relative to the FITC protons H^1^ and H^2^, 4H) suggests that the FITC/G4 PAMAM ratio should be 13.5/4 = 3.3.


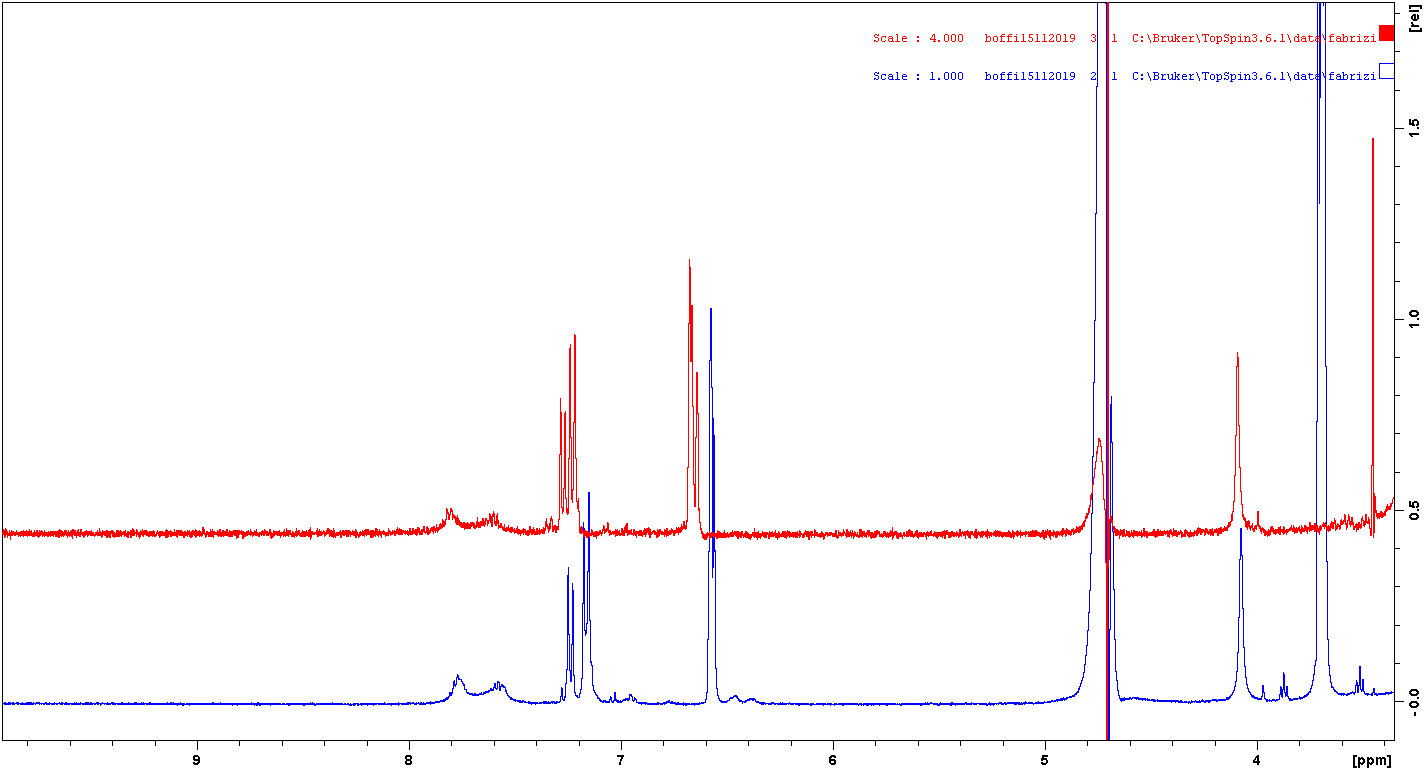


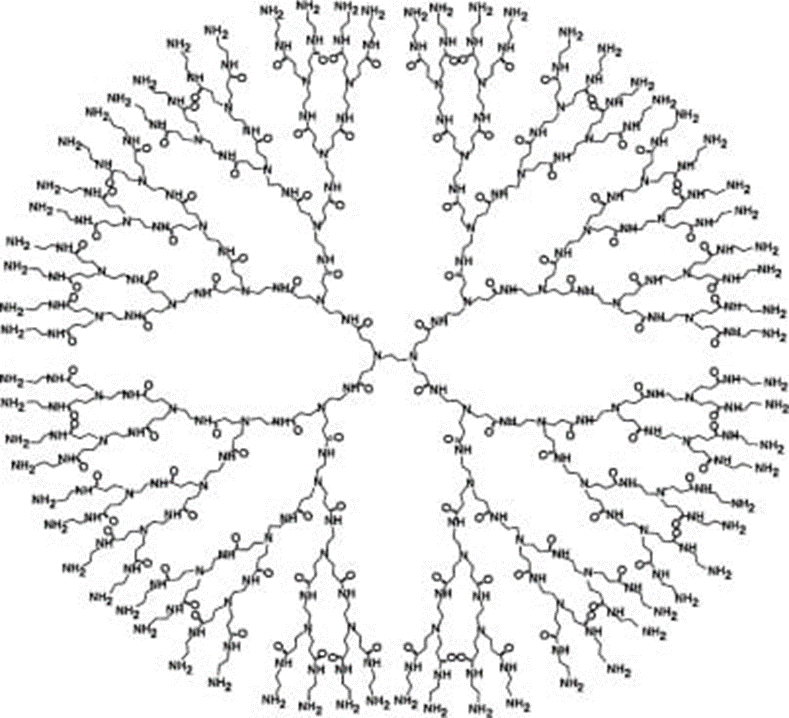


G4 PAMAM structure (498 methylene groups, corresponding to 996 H)

**Figure S5: UV-vis and HP-SEC analysis of Alexa Fluor 647 labelled HumFt**

Alexa Fluor 647 binding to HumFt was evaluated by UV-vis absorption spectroscopy. The spectrum analysis suggests that the Alexa Fluor 647/HumFt ratio is about 8.

Aggregation state of HumFt-Alexa Fluor 647 was evaluated by HP-SEC analysis, following the fluorescence signal of dyed protein (ex 650 nm, em 665 nm). Attempt to label the protein as a dimer appeared to impair the nanoparticle's ability to assemble properly (red line). This is probably due to the modification of lysine residues involved in the self-assembly process of the 24-meric polymer. When labelling was performed directly on the assembled ferritin, the equilibrium is shifted towards the closed form (blue line). Closed HumFt (24-mer) retention time = 4.5 min; open HumFt (dimer) retention time = 6 min.

.

**Figure S6: HumFt-PAMAM nanoparticle synthesis: free G4 PAMAM removal by PD10 column**

The purification was monitored by HP-SEC at 220 nm. In the experimental condition described in Experimental section, G4 PAMAM elutes at 7.6 min. As shown in figure, a complete G4 PAMAM removal was obtained only after the second PD-10 cleaning step (blue line).

**Figure S7: G4 PAMAM shifts the equilibrium towards closed HumFt (24-mer)**

Mg^2+^ induces HumFt assembly as a 24-mer. In this condition, the open dimeric form is typically about 12% (11.82±2.72%, mean ± SD, n=5). When HumFt is assembled in the presence of Mg^2+^ and G4 PAMAM, dimer percentage significantly lowers to 2.34 ± 0.85% (n=5, t-test, p<0,001), indicating that G4 PAMAM shifts the equilibrium towards the closed form.

**Figure S8: HumFt-G4 PAMAM nanoparticle stability**

HumFt-PAMAM nanoparticle stability was tested up to 24 hours at 37°C in i) 20 mM HEPES buffer containing 50 mM MgCl_2_, ii) PBS buffer, and iii) RPMI 1640 medium containing 10% FCS. In all the tested condition the hybrid nanoparticle is stable, being the HumFt 24-mer always higher than 95%. In figure are shown representative chromatograms of HumFt-PAMAM nanoparticle in RPMI 1640 medium with 10% FCS (panel A) and in PBS (panel B) after 24 hours incubation at 37°C, both compared with HumFt-PAMAM in HEPES and MgCl_2_ (red chromatogram). The measurements were performed in triplicate and the results are reported as mean ± SD (panel C).

**Figure S9:** **FACS analysis of NB4 cells treated with HumFt-PAMAM hybrid nanoparticles**

NB4 cells, incubated for 24 hours with HumFt-Alexa Fluor 647 labeled (red), or G4 PAMAM-FITC labeled (green), or a complex of the two labeled molecules were analyzed by flow cytometry. On the left the forward/side scatter dot plots are shown, indicating that none of the compounds affects cell viability or physical properties like size and internal complexity (granulosity). HumFt is uptaken by the entire cell population, as well as PAMAM. Taking into consideration the data obtained by confocal microscopy (see manuscript Figure 4), we hypothesized that the less intense green signal of the double labelled nanoparticle may be due to a quenching effect of the protein shell.

**Figure S10: Morphological analysis of NB4 cells treated with HumFt-PAMAM-miR145 hybrid nanoparticle**

Multiple fields at smaller magnification of the morphological analysis shown in Figure 6B.

**Figure S11: Proliferation and viability of NB4 cells treated with HumFt-PAMAM-miR154 hybrid nanoparticle**

**A)** NB4 cells, incubated for 24 hrs with a complex of HumFt-FITC labelled-PAMAM-pre-miR145 or with HumFt-PAMAM-pre-miR145 were analyzed by flow cytometry to evaluate the uptake efficiency of the complex. The left panels show the flow cytometry histogram indicating the intensity of FITC fluorescence incorporated into the cells, the right panel reports the mean fluorescence intensity of biological triplicate samples (n=3 ± SEM). The data shows that the complex is internalized by the entire cell population.

**B)** NB4 cells, incubated for 24 hrs or 48 hrs with HumFt-PAMAM-pre-miR145 nanoparticle, were counted by trypan blue exclusion assay (left panel), and analyzed by flow cytometry (right panel) upon staining with propidium iodide to evaluate cell viability (n=3 ± SEM).
